# Development and Applications of the Copper-Catalyzed Azide-Alkyne Cycloaddition (CuAAC) as a Bioorthogonal Reaction

**DOI:** 10.3390/molecules21101393

**Published:** 2016-10-24

**Authors:** Li Li, Zhiyuan Zhang

**Affiliations:** 1 School of Life Sciences, Peking University, Beijing 100871, China; lili0224@nibs.ac.cn; 2 National Institute of Biological Sciences, Beijing 102206, China

**Keywords:** CuAAC, click reaction, bioorthogonal reactions, imaging, activity-based protein profiling

## Abstract

The emergence of bioorthogonal reactions has greatly broadened the scope of biomolecule labeling and detecting. Of all the bioorthogonal reactions that have been developed, the copper-catalyzed azide-alkyne cycloaddition (CuAAC) is the most widely applied one, mainly because of its relatively fast kinetics and high efficiency. However, the introduction of copper species to in vivo systems raises the issue of potential toxicity. In order to reduce the copper-induced toxicity and further improve the reaction kinetics and efficiency, different strategies have been adopted, including the development of diverse copper chelating ligands to assist the catalytic cycle and the development of chelating azides as reagents. Up to now, the optimization of CuAAC has facilitated its applications in labeling and identifying either specific biomolecule species or on the omics level. Herein, we mainly discuss the efforts in the development of CuAAC to better fit the bioorthogonal reaction criteria and its bioorthogonal applications both in vivo and in vitro.

## 1. Introduction

For the better understanding of living systems, scientists have made every endeavor to monitor biomolecules in their native context. Among all the visualization and probing strategies, the development of fused fluorescent protein such as GFP and its variants has become the most widely applied one and has had great impact [1,2,3]. Undoubtedly, the successful fusion of a fluorescent tag to a certain protein enables its precise imaging and tracking in living cells and even organisms. Powerful as it is, fusion of fluorescent protein tags still has limitations. Biomolecules other than proteins cannot be genetically modified to attach such fluorescent tags. Also, the addition of a fluorescent protein domain might perturb the structure and function of proteins due to its relatively large size. Another point to mention is that fluorescent protein tags can only be attached to known proteins for detection, but can’t provide guidance during phenotype instructed studies. To overcome these restrictions, bioorthogonal reactions have emerged as an alternative option for monitoring and probing cellular processes.

**Figure 1:**
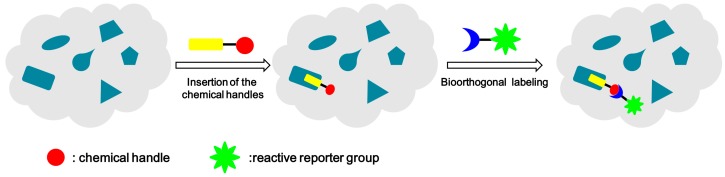
Bioorthogonal strategy for biomolecule labeling.

Labeling biomolecules through bioorthogonal reactions is typically performed in a two-step process. First, a chemical handle, which is also called the reporter group, is incorporated into the biomolecules of interest; next, reactive probes with tags for detection or purification are added to the system and react in situ with the reporter group (Figure 1). Bioorthogonal reactions can overcome the limitations of fluorescent protein labeling. The installation of the reporter group can be achieved through metabolic incorporation, enzymatic labeling, and activity-based covalent binding of probes [4]. These methods extend the scope of labeling to non-portentous biomolecules including glycans, lipids, nucleic acids and other metabolites [4,5,6,7]. Besides, the reporter groups and the reactive probes are usually small chemical moieties, and would cause much less functional and spatial interference than the fluorescent protein domain.

To successfully perform the ligation step and minimize the influence to stability and homeostasis of the probed cells or organisms, bioorthogonal reactions must meet stringent requirements: (i) the reaction between the two reactive components must be highly selective, and meanwhile not affect the vastly diverse biomolecules and metabolites in the surrounding environment; (ii) the reaction must proceed efficiently and rapidly in an aqueous environment under physiological conditions (pH = 6~8, temperature = 37 °C, while exposed to O_2_); (iii), all reaction components, including the reagents and products, should be biocompatible and non-toxic to cells or organisms; (iv), the functional groups should be as small as possible in order to avoid possible perturbations to the labeled biomolecules.

**Scheme 1:**
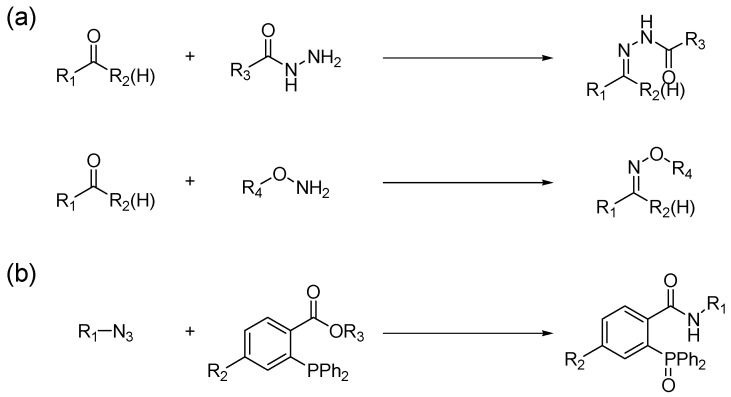

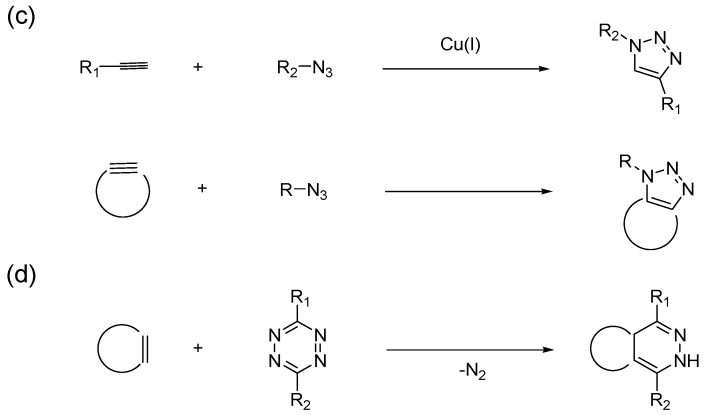
Examples of bioorthogonal reactions. (**a**) ketone/aldehyde condensation; (**b**) Staudinger reaction; (**c**) 1,3 bipolar cycloaddition; (**d**) Inverse-electron demand Diels-Alder reaction.

Under such rigorous criteria, only a handful of chemical reactions stand out as qualified bioorthogonal reactions. These reactions include, but are not limited to lysine/cysteine modification reactions (such as Michael addition, thiol-ene reaction, and so on), the ketone/aldehyde condensation, Staudinger reaction, 1,3-dipolar cycloaddition, and the inverse electron demand Diels-Alder reaction, (Scheme 1) [7,8,9].

Lysine/cysteine modification reactions could achive the bioconjugation on specific amino acid residues of proteins. Lysine can be labeled with activated esters, sulfonyl chlorides, isocyanates, or isothiocyanates [8]; cysteine can undergo disulfide exchange, Michael addition and photoinduced thiol-ene click reactions [8,10]. These reactions all deal with existing functional groups in living systems, and therefore were mainly performed during purified protein labeling [8,11].

Ketone/aldehyde condensation with aminooxy/hydrazide compounds was the first explored bioorthogonal reaction two decades ago (Scheme 1a) [12].

This reaction was found to proceed in aqueous solution in an optimal pH range from 5 to 6 [13]. The ketone group was incorporated in the sialic acid residues on cell surface through a metabolic pathway and successfully labeled by aminooxy- and hydrazide-linked probe molecules [12,14,15]. However, further applications of this reaction inside cells or organisms were hindered by several aspects. Ketone/aldehyde condensations have rather slow reaction rates, with second order rate constants (k_2_) between 10^−4^ to 10^−3^ M^−1^·s^−1^ [16]; the optimum pH of this reaction is not achievable in vivo; the issue of side reactions with the abundant keto- and aldehydic metabolites inside cells is also another hindrance [4].

The next investigated was the Staudinger ligation of azides with functionalized phosphines. This was modified from the classic Staudinger ligation by introducing an intramolecular trap in the phosphines (Scheme 1b) [17]. Both azides and phosphines are absent from biological systems. The azido group has a small size, remains stable and inert under physiological conditions, thus making it suitable to be incorporated as the chemical handle [4]. Staudinger ligation has been applied in labeling glycans both in cells and organisms [17,18,19]. Unnatural amino acids (UAA) bearing azido groups have been developed and incorporated into proteins for phosphine labeling [20,21]. Although Staudinger ligation has clear advantages over the ketone/aldehyde condensation reaction, it still suffers from slow reaction kinetics. The second-order rate constant of this reaction was reported to be within the range of 10^−3^ to 10^−2^ M^−1^·s^−1^ [22]. While the azido group is an suitable bioorthogonal component, the oxidation of the phosphine reagents by air or metabolic enzymes is another problem for performing Staudinger ligation in vivo [9].

The applications of ketone/aldehyde condensation and Staudinger ligation were focused on labeling cell surface glycans [12,14,15,17,18,19] and purified proteins [20,21]. This has already extended the monitoring scope beyond the limitation of proteins, and revealed the huge potential of bioorthogonal reactions in labeling and detecting various kinds of biomolecules. However, the early explorations also indicated that difficulties remained in expanding their applications in vivo, and this is mainly due to the slow reaction rates. Neither of the two reactions have second-order rate constants larger than 10^−2^ M^−1^·s^−1^ [16,22]. Therefore, in order to achieve a sufficient amount of labeling, high concentrations of reactive probes and long reaction times are required, which would put too much pressure on cells.

In the meantime, an alternative reaction of azide came to the light, that is, the copper-catalyzed azide-alkyne cycloaddtion (CuAAC, Scheme 1c) [23,24]. It had already been verified that the azide moiety is a powerful chemical reporter group; the alkyne group also has same properties: small size, stability and absence from biological systems. Compared with other bioorthogonal reactions, CuAAC has better kinetics and reaction efficiency and greatly reduces the reaction time and reagent concentrations [23,24,25].

Inverse-electron demand Diels-Alder reactions (IED-DA) were demonstrated to be bioorthogonal in 2008 by the Fox group [26] (Scheme 1d). These reactions between tetrazines and strained alkenes or alkynes are the fastest bioorthogonal reactions to date, with rate constants between 10^3^ and 10^6^ M^−1^·s^−1^ [5,9]. IED-DA reactions have been exploited for labeling and detecting various kinds of biomolecules in vivo and in vivo [5].

Among all the developed bioorthogonal reactions, CuAAC is the most powerful bioorthogonal reactions up-to-date, though the cytotoxicity caused by the introduction of Cu(I) ions casts a shadow over its in vivo application prospects [27]. Enormous efforts have been made to minimize or avoid Cu(I) ion-induced cytotoxicity and further optimize the kinetics and efficiency. These efforts include developing copper stabilizing ligands to assist the reaction, developing chelating azide as reagents, and using ring strain instead of a copper catalyst as the driving force. Here, we summarize and discuss all these efforts in the development of CuAAC and its applications as a bioorthogonal reaction for biomolecule labeling and detection.

## 2. History of CuAAC

**Scheme 2:**
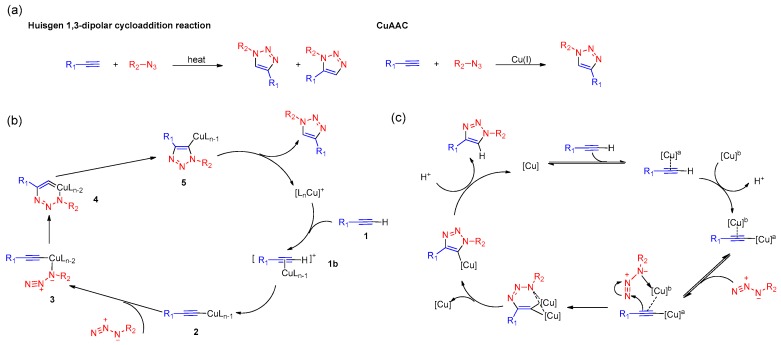
CuAAC reaction and the proposed mechanism. (**a**) CuAAC reaction; (**b**) Early mechanism proposal by Sharpless; (**c**) Mechanism proposal with a dinuclear copper intermediate.

CuAAC is a variant of the Huisgen 1,3-dipolar cycloaddition reaction (Scheme 2a), which has been applied in organic chemistry for five-membered heterocycle synthesis ever since the 1960s [28]. The formation of 1,2,3-triazole rings by azide-alkyne cycloaddition was among the most useful reactions in this family. However, the uncatalyzed reaction often requires elevated temperature and a long reaction time, and has a problem of poor regioselectivity unless with highly electron-deficient terminal alkynes are used as reagents [28].

Various endeavors have been made to control the regioselectivity to specifically produce 1,4- or 1,5-regioisomers, and this has been successful to different extents [29,30,31,32,33,34]. The most effective breakthrough strategy was the discovery of Cu(I) ion catalysis independently reported by the Sharpless and Meldal groups in 2002 (Scheme 2a) [23,24]. Cu(I) catalysis not only dramatically improved the regioselectivity to solely afford the 1,4-regioisomer, but also accelerated this reaction to as much as 10^7^ times faster than the uncatalyzed version so that ambient temperature was enough to drive the reaction. Apart from exclusive regioselectivity, fast kinetics and high yield, the following other improvements were also observed in the reports of the two groups: tolerance of a wide scope of various reagent structures, insensitivity to the usual reaction parameters such as pH level (4–12) and solvents (H_2_O compatible), avoidance of functional group interference, and convenient in situ preparation of Cu(I) ions [23,24]. Actually, all these advantages of CuAAC could meet the criteria of click reactions, and furthermore, qualified it as a potential bioorthogonal reaction.

## 3. Mechanistic Studies of CuAAC

In order to illustrate the fantastic properties of CuAAC, mechanistic investigations of CuAAC have been performed through computational calculation, kinetic studies, real-time reaction monitoring, and isotope exchange methods [35,36,37].

Along with the discovery that Cu(I) ions could regioselectively catalyze azide-alkyne cycloadditions, the Sharpless group proposed a stepwise mechanism (Scheme 2b) with a monomeric copper(I) acetylide complex as the intermediate in the catalytic cycle [23]. This proposal is quite different from the concerted mechanism of the uncatalyzed cycloaddition. The stepwise mechanism was further supported by a density functional theory (DFT) study, which also helped explain the regioselectivity, the preference for an aqueous environment and the fast kinetics [35]. In the formation of copper(I) acetylide, the displacement of various ligands from the copper(I) center by the alkyne varied greatly. Calculation results showed that when the ligand was H_2_O, this process was exothermic by 11.7 kcal/mol, more favorable than the 0.6 kcal/mol endothermic calculation result with acetonitrile as the ligand. Based on the uncatalyzed thermal cycloaddtion, the activation barriers calculated for 1,4- and 1,5-regioisomers of the 1,2,3-triazole were 25.7 and 26.0 kcal/mol, respectively. The close activation barriers could account for the usual formation of regioisomer mixtures without Cu(I) ions as catalyst. The barriers for a concerted mechanism involving species **1b** or **2** (Scheme 2b) to form the 1,4-regioisomer were also calculated, but neither of the results (27.8 kcal/mol for **1b** and 23.7 kcal/mol for **2**) could explain the fast kinetics. However, the calculation for the activation barrier of the intermediate **3** to **4** (Scheme 2b) in the stepwise mechanism was calculated to be 14.9 kcal/mol, much lower than that of the uncatalyzed reaction and sufficient to explain the 10^7^× rate enhancement [25].

While DFT calculations were performed in terms of the supposed stepwise mechanism, the presumed intermediate is not in agreement with later work, making the calculations inapplicable in that context. The dependence of rates on concentrations in the reaction of benzyl azide and phenylacetylene are consistent with a stepwise mechanism that involves a dynamically exchanging family of Cu-acetylide complexes [36]. One noteworthy thing was that the kinetic studies indicated that under the conditions of Cu(I) catalysis and saturating alkynes and azides, the reaction was second order in copper. This indicated that either two metal centers were responsible for the activation of azide and alkyne, respectively, or a multinuclear Cu-acetylide was involved. Direct evidence for a dinuclear copper intermediate was later provided through the method of real-time monitoring of the reaction progress and TOF-MS analysis of isotope crossover studies [37]. Reaction progress monitoring showed that the prepared mononuclear σ-bound copper(I)-acetylide was unreactive towards azide without the addition of exogenous copper catalyst. Further investigation into the role of each copper atom by TOF-MS analysis of isotope crossover led to the proposal of a ligand exchange process in the intermediate (Scheme 2c). The DFT calculation of reaction barriers for cyclization of the dinuclear copper intermediate was recently reported [38].

Despite all the progress in illustrating the mechanism underlying CuAAC, there still exists much controversy. The step of azide chelation to the copper center was always considered the rate determining step in the whole catalytic center, but the details was not clearly explored [39]. Also, the proton source for the protonation of the triazole-copper intermediate to afford the final product remains unclear. Further studies into the very nature of this reaction are still needed.

## 4. Early Bioorthogonal Applications of CuAAC and Its Limitations

Soon after the discovery of Cu(I) catalysis of azide-alkyne cycloaddtion, CuAAC became the best click reaction, and found wide applications in organic synthesis [34], combinational chemistry [40], drug development [41], material science [42], etc. Also, its potential as a bioorthogonal reaction has been rapidly verified in several aspects [43].

The first successful bioorthogonal exploitation of CuAAC was the bioconjugation on the exterior surface of Cowpea Mosaic Virus (CPMV) [44]. The reactive lysine or cysteine residues were decorated with azides or alkynes, and then ligated with fluorescein derivatives with the complementary reactive groups. All 60 identical protein units on CPMV capsid were fluorescently labeled. Another notable point was the addition of tris(triazolyl) amine (TBTA) as a ligand, which greatly raised the reaction rate, and stabilized the Cu(I) oxidation state in aqueous solution.

Exploration of CuAAC as a bioorthogonal reaction was further carried on following this first example. *E. coli* outer membrane protein C (Omp C) was selectively labeled by TBTA-assisted CuAAC after metabolic incorporation of the azido functional group using azidohomoalanine as a methionine surrogate [45,46]. The tris(triazolyl) amine ligand TBTA could enhance the CuAAC reaction efficiency, but its poor solubility in water set a limitation on reagent concentration. Other than metabolic incorporation to introduce azide or alkyne groups, the Schultz group developed the genetic incorporation of azide or acetylene containing unnatural amino acids and demonstrated its efficiency by site-specific CuAAC labeling of human superoxide dismutase-1 (SOD) protein [47].

Apart from biomolecule labeling and imaging, CuAAC also allowed a huge advance in the activity-based protein profiling (ABPP) method, which employs active site directed chemical probes to profile their selective target proteins on a whole proteomic level [48]. Previous probes with tags for enrichment or visualization were usually bulky due to the biotin/fluorophore moieties. This limited their cellular uptake and distribution, and might have a large influence on their activity, so the ABPP method used to be performed in cell or tissue homogenates rather than in the native cellular environment. To overcome these restrictions, the Cravett group developed the CuAAC-assisted ABPP method [49,50]. The merit of this advanced ABPP method lied in the uncoupling of the proteome labeling and tag addition into two separate steps. The activity and cell permeability of active probes carrying an azide or alkyne group instead of a bulky tag moiety are less affected and therefore could be exploited in vivo. The CuAAC ligation is highly selective and assured the enrichment or visualization tags being exclusively ligated to the probe-modified proteins. CuAAC-assisted ABPP was successfully exploited in profiling the enzyme targets of azide-derived phenyl sulfonate probes both in the living cell and in mice [49,50]. Higher level of background labeling compared with standard ABPP was reported, but was later circumvented by using the alkyne version of the same probe [50].

In these early stage utilization examples, the power of CuAAC as a bioorthogonal reaction was clearly demonstrated. Nevertheless, the limitations were also revealed at the same time. The first problem is that efficiency of CuAAC drops greatly compared with when conducted in an organic solution environment, which necessitates the addition of the tris(triazolyl) amine ligand TBTA [44]. TBTA can enhance the reaction rate and yield in buffer or medium, but it suffers from poor solubility [45].

Another problem is the side reactions. Under organic conditions, by-products such as diynes, bistriazoles, and 5-hydroxytriazoles were observed [23]. When applied in buffer condition, non-specific reactivity was reported both in surface labeling and the ABPP method with excessive amount of alkyne reagents [45,49]. Diynes were formed as by-products from Cu(II) catalyzed oxidative coupling of terminal alkynes [44].

The most severe problem that needs to be considered is the toxicity caused by copper ions. Copper ions can be easily chelated by native amino acid residues to damage the structure and function of proteins, and can induce the formation of reactive oxygen species (ROS) [27,51,52]. *E*. *coli* cells subjected to CuAAC ligation on the cell surface were unable to divide after transfer back to rich medium [45]. Above micromolar concentration copper ion will cause severe cellular damage or even death [27]. Besides the toxicity caused by Cu(I) species, high levels of triazole products with the existence of Cu(II) could cause decomposition and degradation of biomolecule complexes such as CPMV capsid [44].

Taking these three major limitations into consideration, though CuAAC proved its orthogonality and efficiency in biological systems, very few early application examples were performed inside cells. To further broaden the application scope of CuAAC as a bioorthogonal reaction in living systems, these obstacles have to be surmounted. Optimization of CuAAC has therefore been focused on the achieving the following goals: higher reaction rates, higher yields, less side reactions and less toxicity.

The core of the cytotoxicity problem lies in the excess amount of Cu(I) species taken up by cells, and the simplest solution would be to reduce the Cu(I) concentration. However, a sufficient amount of Cu(I) is required for maintaining the reaction rate and suppressing side reactions. Over the last decade, three distinct strategies have been developed to deal with this Cu(I) dilemma and optimize CuAAC reaction: (1), ligand-assisted CuAAC; (2), strain-promoted Cu(I)-free azide-alkyne cycloaddition (SPAAC); and (3), chelation-assisted CuAAC [6,9]. These strategies together with their applications in bioorthogonal labeling and imaging are discussed in details in the following sections.

## 5. Ligand-Assisted CuAAC and Its Applications as a Bioorthogonal Reaction

### 5.1. Development of Different Classes of Ligands

**Scheme 3:**
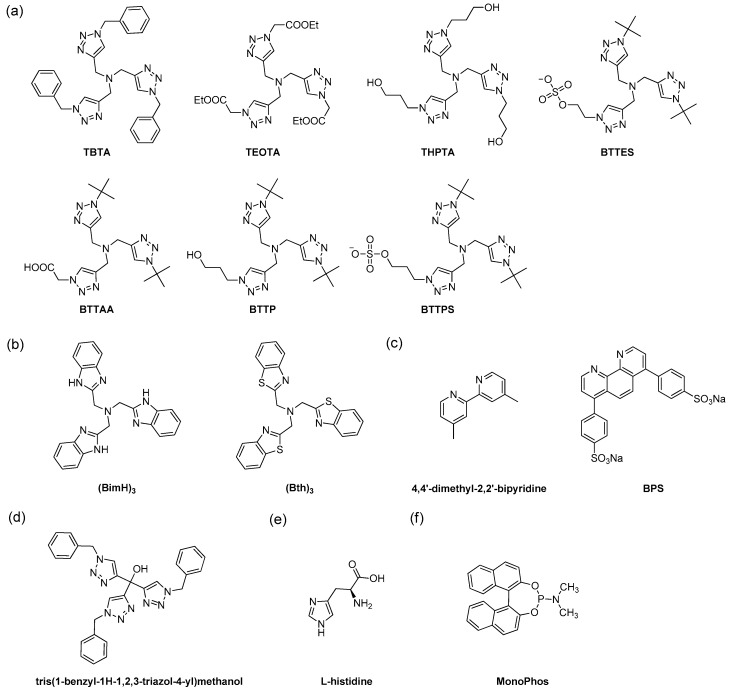
Accelerating ligands for CuAAC reactions: (**a**) TBTA and its analogues; (**b**) tris(heteroarylmethyl)amine ligands; (**c**) 2,2′-bipyridine and 1,10-phenanthroline derivatives; (**d**) tris(1-benzyl-1*H*-1,2,3-triazol-4-yl)methanol; (**e**) L-histidine; (**f**) *P*-donor ligands.

The first applied ligand in CuAAC was TBTA [44] (Scheme 3a). Before its formal publication, TBTA has been used in various examples as mentioned above to enhance the efficiency of CuAAC as a bioorthogonal reaction. Improvements in reaction rate and yield with the assistance of TBTA were demonstrated in all these studies [44,45,46,47,49,50].

In 2004, the Fokin group reported that polytriazoles could function as ligands in CuAAC, and compared a series of polytriazoles to find out that TBTA worked the best [53]. In cyclic voltammetry studies a dramatic increase in the redox potential of Cu(I)/Cu(II) was observed when TBTA was added, indicating that TBTA could stabilize the Cu(I) oxidation state. This was proposed to result from the tetradentate binding ability that could block all possible coordination sites for potential oxidant attack [53]. The basic tertiary amine structure was also supposed to accelerate the catalytic process by providing additional electron density for the Cu(I) center [53]. However, in the crystal structures of TBTA coordinated with Cu(I) and Cu(II) resolved later, the basic tertiary amine binds with the Cu(II) atom, but not the Cu(I) atom [54]. The Cu(I)-TBTA complex was an unusual dinuclear dication: each of the two Cu(I) atoms is coordinated with the proximal *N* atom of the triazoles in one TBTA, and the medial *N* atom of a triazole from the other bridging TBTA. Besides TBTA, bistriazoles also showed strong ligand accelerating effect, but their effect drops greatly when the amount of Cu(I) catalyst is reduced [53].

TBTA has facilitated the utilization of CuAAC in biological fields for its efficiency enhancement and catalyst stabilization effects, but it suffers from poor water solubility, and doesn’t help with the cytotoxicity issue. Luckily, inspired by TBTA, studies of catalytic ligands with various structures soon followed, and some of them were mainly focused on improvement of the biocompatibility issues.

Based on the tetradentate ligand structure of TBTA, researchers kept the tertiary amine and three triazole rings, and optimized around the benzyl rings to further improve the water solubility and Cu(I) stabilization properties for better biocompatibility. This class of ligands includes THPTA [55], BTTAA [56], BTTES [57], TEOTA [58], BTTP, BTTPS [59], etc. (Scheme 3a, discussed in details later). A similar class of ligands are the tris(heteroarylmethyl)amine group (Scheme 3b) [60]. While the central tertiary amine structure remains the same, nitrogen heterocylces are used for association instead of triazole rings. Typical examples are tris(2-benzimidazolylmethyl)amine ((BimH)_3_), tris(benzothiazole)amines ((Bth)_3_), and mixed benzimidazole/benzothiazole ligands ((BimH)_2_(Bth) and (BimH)(Bth)_2_). N substitution of the tris(benzimidazole) ligand by small pendant alkyl and ester groups ((BimC_1_E)_3_, (BimC_1_E’)_3_ and (BimC_1_H)_3_, not listed here) was explored as well. This class of ligands is superior to TBTA as catalytic ligands in accelerating CuAAC reactions [60,61,62].

2,2′-Bipyridine and 1,10-phenanthroline derivatives were also found to be effective ligands for CuAAC through a screening method (Scheme 3c) [63]. Increasing the electron density on the aromatic rings of 2,2′-bipyridine can afford more rapid reaction rates than possible with bipyridine itself. A library of Schiff bases were screened using the same method, and the screen indicated Schiff bases containing a pyridine group in a suitable position to form a chelating interaction could work as accelerating ligands. Among this class of ligands, bathophenanthrolinedisulfonic acid (BPS) has the best rapid CuAAC acceleration properties [63]. It is superior to TBTA in water solubility and works more effectively in accelerating CuAAC reactions on the CPMV surface, but showed poorer Cu(I) stabilization than TBTA [64].

To improve the water solubility, tris(1-benzyl-1*H*-1,2,3-triazol-4-yl)methanol was developed as a ligand for CuAAC (Scheme 3d) [65]. By replacing the tertiary amine N atom of TBTA with a C atom and adding a hydroxyl group, this ligand has complementary hydrophobic and hydrophilic characters, and its complex with CuCl was highly active in CuAAC reactions under aqueous conditions [65,66].

The natural amino acid l-histidine can also effectively facilitate CuAAC as a copper ligand (Scheme 3e), and it was reported that the Cu(I)-l-histidine complex exhibited the least toxicity compared with TBTA, BPS and THPTA on four different cell lines, which indicated its exploitation for inside cell CuAAC [27].

Besides *N*-donor ligands, *P*-donor ligands were also explored (Scheme 3f). Phosphoramidite ligands (e.g., MonoPhos) were shown to accelerate CuAAC reactions under aqueous conditions [67]. Triphenylphosphine (PPh_3_) were also shown to accelerate CuAAC, and at the same time function as a reductant, but the Staudinger side reaction was a concern [68]. The efficiency of *P*-donor ligands has not been compared with TBTA.

### 5.2. Kinetic and Mechanism Studies of Ligand Assisted CuAAC

Reaction kinetics of *N*-donor ligand-assisted CuAAC have been systematically investigated by the Fokin and Finn groups. They found a second order dependence on copper for most of the ligands, and a “threshold” point for the ligand/Cu ratio, which suggested there might be more than one mechanism operating under the detected conditions [61,62]. Investigation of other factors showed that other than ligand/Cu ratio, the presence of chloride ions and different types of reaction solvents also affected the reaction kinetics [61,62]. However, TBTA is an exception. A first order rate dependence was observed in the presence of TBTA, and the reaction kinetics were continuous without a “threshold” point [61].

For the investigated ligands TBTA and (BimH)_3_, the rate order in azide was influenced by the addition of chloride ions. The reactions were almost zero-order in azide in water, and a first-order dependence on azide was observed in the presence of chloride ions [61]. The sensitivity of rate order in azide suggests that Cl- and azide have similar affinities for coordinating with Cu(I), and that Cu(I)-N_3_ interaction might be a significant step in the ligand-assisted CuAAC catalytic cycle [61].

### 5.3. Biocompatible Ligands

Apart from sharing the common property of improving CuAAC kinetics, biocompatible ligands that can be used to assist CuAAC as a bioorthogonal reaction should have other important features as preferable water solubility, strong stabilization of the Cu(I) oxidation state, reduction of Cu-induced cytotoxicity, and lower copper usage. Of the abovementioned CuAAC ligands, TBTA, THPTA, BTTAA, BTTES, TEOTA, BTTP, BTTPS, BPS and l-histidine have been applied in bioorthogonal CuAAC. THPTA, BTTES and BTTAA are the most biocompatible ones.

To overcome the vexing problems revealed by the early bioorthogonal applications using TBTA as the ligand, the Finn group developed the more water-soluble ligand THPTA as the revised version of tris(triazolylmethyl)amine ligand. THPTA could accelerate the CuAAC reaction better than TBTA. More importantly, at a ligand/Cu ratio of 5:1, THPTA could protect the histidine moiety from oxidation, lower the ROS levels induced by Cu(I) ion, and avoid protein crosslinking [55]. All these optimized properties enable its applications in living systems.

BTTES and BTTAA are other TBTA derivatives developed by the Wu group. BTTES bears two *tert*-butyl groups to enhance its activity and a sulfate functionality to minimize the cell membrane permeability of the coordinated copper and at the same time balance its solubility. At a ligand/Cu ratio of 6:1, BTTES-Cu complex showed almost no cellular toxicity effects, and no long-term perturbation of cells [57]. BTTP and BTTPS are BTTES analogues with similar properties [59]. BTTAA was developed by replacing the sulfate functionality of BTTES with an acetic acid group, which not only bears a negative charge, but also serves as an additional weak donor to coordinate with Cu(I) ions to achieve additional acceleration. BTTAA-Cu(I) complex also showed neither cellular toxicity nor long-term perturbation [56]. BTTEA and BTTAA are more suitable ligands for cell out surface labeling compared with TBTA and THPTA [6].

### 5.4. Bioorthogonal Applications of Ligand Assisted CuAAC

**Figure 2:**
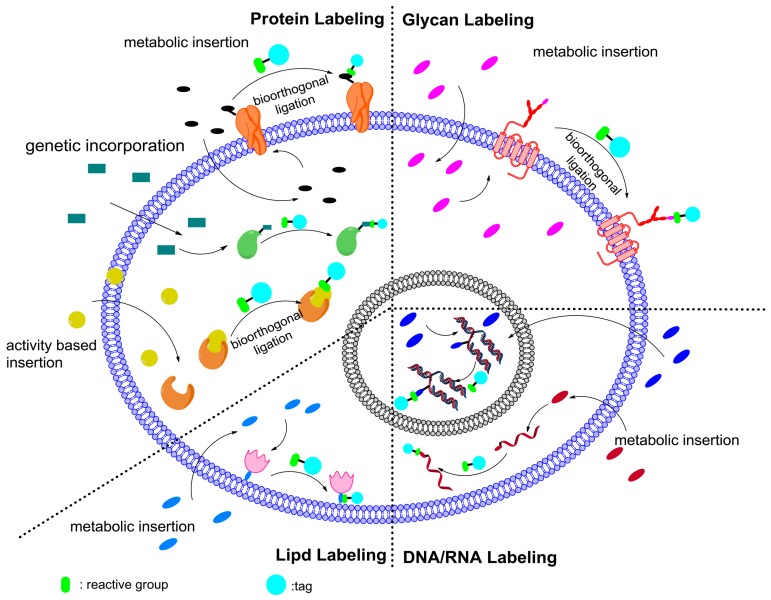
Bioorthogonal labeling of biomolecules with CuAAC.

Taking advantage of biocompatible copper ligands, applications of CuAAC as a bioorthogonal reaction in labeling and detection has been extended to almost all classes of biomolecules, including proteins [45,46,69,70,71,72,73,74,75], glycans [56,57,59,76,77,78,79,80,81,82], lipids [83,84,85], DNA [86,87], RNA [88] etc., either for “omics” studies or for tracing specific biomolecules (Figure 2). To explore biological systems using CuAAC, alkyne or azide chemical handles should firstly be inserted into the investigated biomolecules; then the reactive probe molecules with the corresponding functional group will be added to the system, in the presence of a suitable ligand-Cu complex and reducing agent. Studies have shown that reactive probe molecules bearing azides yield cleaner results than those bearing alkynes, since alkynes are more prone to side reactions [44,50].

#### 5.4.1. Protein Labeling

Chemical handles can be incorporated into proteins through metabolic insertion [45,46,69,70], unnatural amino acid mutagenesis systems [71,72,73] and activity-based incorporation [74,75] (Figure 2). The first method is commonly used for labeling the whole proteome, while the latter two can be applied in labeling specific proteins.

Using azidohomoalanine (AHA) as a metabolic analogue of methionine, the Tirrell group successfully labeled the surface of *E. coli* cells with TBTA as a ligand [45,46]. Later, by metabolic incorporation of alkyne-bearing unnatural amino acids like homopropargylglycine (HPG), ethynylphenylalanine (ETH), and homopropargylglycine (Hpg), they labeled newly expressed protein inside both *E. coli* cells and mammalian cells by means of in situ fluorescence generation [69,70].

Site-specific incorporation of chemical handles is usually achieved by using the unnatural amino acid mutagenesis system with the help of an orthogonal aminoacyl-tRNA synthetase (aaRS)-tRNA pair. Along the years, various unnatural amino acids bearing alkyne or azide groups have been developed and applied in specific protein imaging through CuAAC. An azide-bearing pyrrolysine analogue, *N*^ε^-(((1*R*,2*R*)-2-azidocyclopentyloxy)-carbonyl)-l-lysine (ACPK), was incorporated into HdeA-V58-3 protein in *E. coli* and p53EGFP-K372-3 protein of the mammalian cell line HEK293T using BTTES-assisted CuAAC [72]. ACPK was also specifically incorporated into the T3S effector protein OspF in *Shigella*, and subsequently labeled with a biotin tag through TBTA-assisted CuAAC [71]. This facilitated the detection of the *Shigella* toxin without disruption of its enzymatic activity. More recently, a multifunctional unnatural amino acid, N_3_-p-azidobenzyloxycarbonyl lysine (PABK), was genetically encoded into engineered EGFR protein on the HEK293T cell membrane. BTTAA-assisted CuAAC was successfully performed between this EGFR-N128PABK-EGFP protein and alkyne-Cy5 on live cells and high fluorescence co-localization was achieved [73].

Activity-based incorporation of chemical handles depends on the availability of azide- or alkyne-bearing active small molecules that can covalently bind with their target proteins. The active small molecules can either be enzyme substrates or artificially developed probes. The alkyne-bearing *N*-but-3-ynyl-2-chloroacetamidine (NoX) can selectively and covalently bind with the enzyme dimethylarginine dimethylaminohydrolase isoform 1 (DDAH-1). After in vivo incubation, NoX-bound DDAH-1 could be fluorescently labeled and detected through TBTA-assisted CuAAC [74]. Another example was the labeling and detection of protein tyrosine phosphatases (PTPs). Alkyne-bearing redox-based probes selectively bind with PTPs in living cells, and BTTP-assisted CuAAC was applied for the subsequent monitoring [75].

#### 5.4.2. Glycan Labeling

Glycans are important biomolecules responsible for mediating cell surface recognition processes [89], intracellular trafficking [90], and transcriptional regulation [91,92]. Changes in glycosylation are related to malignant transformation and the development of a chronic inflammatory states at a cellular level [93,94]. Glycans cannot be genetically monitored, but bioorthogonal ligation provides a way for their visualization in their native environments [12,17]. Bioorthogonal chemical handles are incorporated into glycans through functional metabolic precursors using endogenous biosynthetic pathways (Figure 2).

ManNAc is the metabolic precursor of *N*-azidoacetylsialic acid (SiaNAz), which is overexpressed on the surface of tumor cells and acts as a tumor marker [95,96]. Azido ManNAc dereivative (Ac_4_ManNAz) could be metabolically inserted onto CHO and Hela cell surface glycoproteins, and then labeled with fluorescence for confocal microscopy investigations through THPTA-assisted CuAAC [78]. Alkynyl ManNAc derivative (Ac_4_ManNAl) were reported to be more efficiently converted into sialic acid than Ac_4_ManNAz by TBTA-assisted CuAAC labeling comparasion, both in cells and in mice [76]. BTTES-, BTTAA-, BTTP- and BTTPS-assisted CuAAC also facilitated the labeling of cell surface glycoproteins on LNCap, Jurkat, HEK293T and other cell lines [56,57,59,77,79]. Applying a *cis*-membrane FRET-based methodology, specific glycoproteins such as integrin αXβ2, EGFR, and TβRI could be visualized by BTTAA assisted CuAAC on live cells [80]. Another functionalized sugar is alkynyl GDP-fucose (GDP-FucAl) used as the metabolic precursor of fucosylated glycans. By metabolic insertion of alkyne groups through GDP-FucAl microinjection in zebrafish embryos and subsequent BTTES- or BTTAA-assisted CuAAC with azido fluorophores, zebrafish embryo morphogenesis was monitored via confocal microscopy [56,57].

Besides cellular imaging, CuAAC has also promoted the studies of glycoproteomics and glycan site mapping. TBTA-assisted CuAAC helped the analysis of sialylated *N*-linked glycoproteins in Ac_4_ManNAl incubated PC3 cells [81]. More than 200 *N*-glycosylation site IDs were inventoried and mapped, of which 68% was not previously documented [81]. Ac_4_ManNAz was inserted in sialyl glycoproteins in healthy and hypertrophic rat hearts, followed by biotin labeling via BTTAA-assisted CuAAC. Proteomic analysis and comparison revealed 21 proteins uniquely identified in the hypertrophic rat hearts and a significant number of sialylated proteins were also upregulated [82].

#### 5.4.3. Lipid Labeling

Lipids play critical roles in cellular events such as enforcing protein membrane binding [97]. Like glycans, lipids also can’t be modified genetically, thus metabolic insertion of bioorthogonal chemical handles into lipids provides a means for their investigation in the native environment (Figure 2).

The alkyne tag was metabolically inserted into choline (Cho)-containing phospholipids using the Cho analogue propargylcholine (propargyl-Cho) [83]. The resulting propargyl-labeled phospholipid molecules were then visualized with high sensitivity and spatial resolution in cells via a CuAAC with fluorophore-labeled azide both in cultured cells and mouse tissues [83]. Alkynyl-fatty acid chemical reporters were developed and metabolically inserted into fatty-acylated proteins. TBTA-assisted CuAAC enabled specific and sensitive fluorescence detection of labeled fatty-acylated proteins in mammalian cells [84]. Using the same method, lipoproteins in *E. coli* were rapidly profiled and analyzed [85].

#### 5.4.4. Nucleic Acid Labeling

Detection of nucleic acids including DNA and RNA is of great importance for monitoring different biological processes (Figure 2). 5-Ethynyl-2-deoxyuridine (EdU) is a metabolic thymidine analogue that could be inserted in DNA during cell proliferation. After EdU incorporation, CuAAC ligation with fluorescent azides and subsequent florescence visualization could be performed in vivo for the investigation of cell proliferation and differentiation [86]. (2′*S*)-2′-Deoxy-2′-fluoro-5-ethynyluridine (F-ara-EdU) is an optimized version of EdU with reduced toxicity that exhibits selective DNA labeling properties. Using F-ara-EdU as a metabolic analogue of thymidine, CuAAC facilitated the detection of DNA synthesis with greater sensitivity [87]. Chemical handle insertion in RNA was also performed after incorporation of azide-modified uridine analogues by transcription reactions. THPTA-assisted CuAAC could label this modified oligoribonucleotides with tags like biotin, and also enabled its functionalization for other applications [88].

#### 5.4.5. Activity-Based Protein Profiling

**Figure 3:**
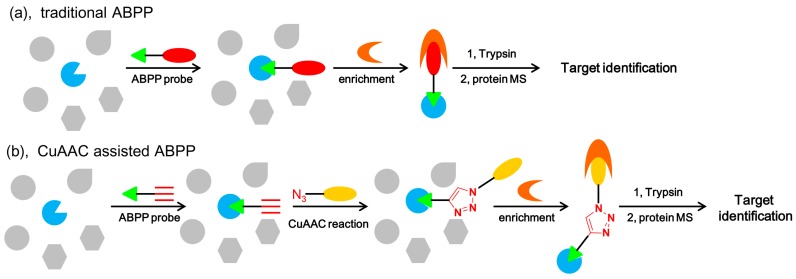
Traditional ABPP (**a**) vs. CuAAC-assisted ABPP (**b**).

As mentioned previously, CuAAC has greatly advanced activity-based protein profiling (ABPP) by uncoupling the proteome labeling and reporter tagging steps of the ABPP process, and therefore circumventing the limitations of bulky tag moieties (Figure 3) [48]. Tag addition by CuAAC reactions has now become a standard procedure in the ABPP method [98]. With this breakthrough, the ABPP method has been widely used in target identification of functional natural products [99,100,101] and phenotypic screening of lead compounds [102], and off-target studies of drugs [103].

Natural products have always inspired the development of drugs and provided research tools for biological studies [99]. Therefore, the target identification of a specific natural product is of great necessity. Since the introduction of CuAAC to facilitate the ABPP method, various classes of natural products have been explored. Probes derived from antibiotics such as penicillin, aztreonam, or cephalosporin were developed and applied in target identification through CuAAC-assisted ABPP methods [100]. These probes revealed the virulence-associated enzyme ClpP and a resistance-associated lactamase as the labeled targets. The target of the anti-cancer natural product duocarmycin was also clarified by CuAAC-assisted ABPP. An alkynyl duocarmycin analogue that maintained its cellular activity was synthesized and applied in ABPP, which subsequently helped the identification of aldehyde dehydrogenase (ALDH1A1) as its binding target [101].

Phenotypic screening identifies active lead compounds that function on a specific phenotype on a cell or organism level. Identifying targets of these lead compounds can promote both the mechanism clarification and related-drug development [104]. Target identification of the neuroprotective chemical P7C3 is a good example of applying a CuAAC-assisted ABPP method for this purpose [102]. Compound P7C3 was selected from a mouse-based phenotypic screen for its potency in fostering the survival of neurons. Its analogue P7C3–S326 containing both a benzophenone for photocrosslinking and an alkyne moiety for CuAAC showed considerable activity and was applied in target identification. By means of CuAAC-assited ABPP, nicotinamide phosphoribosyl-transferase (NAMPT) was identified as its target, and biological experiments showed P7C3 functions through activity enhancement of NAMPT.

Off-target studies are of vital importance for drug development to monitor side effects and toxicity. Based on a proteomic level, ABPP provides as a suitable method for drug off-target studies. Dasatinib is a dual Src/Abl inhibitor for the treatment of imatinib-resistant chronic myelogenous leukemia (CML). Since protein kinases share similar structures at their kinase domain, it is difficult for most kinase inhibitors to display exquisite cellular specificity. A cell-permeable, active dasatinib analogue (DA-2) was developed, which bears both an alkyne group for CuAAC and a diazirine group for photoaffinity labeling. Through CuAAC-assisted ABPP, a number of previously unknown targets of dasatinib were identified, including several serine/threonine kinases (PCTK3, STK25, etc.) [103].

#### 5.4.6. Protein-Protein Coupling

Compared with monomer proteins, hetero- or homo-multimer proteins are widely used in biochemical and medicinal applications for their higher affinity, specificity, and potency for cellular and viral targets [105]. CuAAC has been employed as a protein-protein coupling technique for these protein multimer ligations [105,106].

In 2009, the Tolbert group reported the synthesis of protein G-C37H6 heterodimer via head-to-head CuAAC ligation of two proteins [107]. The alkyne and azide groups were added to the N-terminal of the expressed proteins through native chemical ligation (NCL). The same strategy was also applied to prepare a protein G homodimer and a protein G homotrimer [107]. Since 2009, the Kluger group has been working on the synthesis of hemoglobin bis-tetramers, a hemoglobin-based oxygen carrier, via CuAAC mediated protein–protein coupling [108,109]. An azide group was first introduced onto the β-subunits of hemoglobin, and then an electron-deficient bis-alkyne is used as a linker to produce a fully functional cross-linked hemoglobin bis-tetramer.

Beyond insertion of alkyne or azide handles onto the active residues for protein-protein coupling, site specific protein-protein coupling through CuAAC was achieved by the Swartz group [110]. Using a cell-free protein synthesis system (CFPS), alkyne and azide containing UAAs were incorporated site-specifically into pPaDHFR and pAzGFP proteins, which were directly conjugated at 62% efficiency through TBTA assisted CuAAC [110]. The avoidance of a chemical spacer could maintain the activity of the conjugated heterologous proteins.

## 6. Chelation-Assisted CuAAC and Its Applications as a Bioorthogonal Reaction

### 6.1. Development of Chelation-Assisted CuAAC

**Scheme 4:**
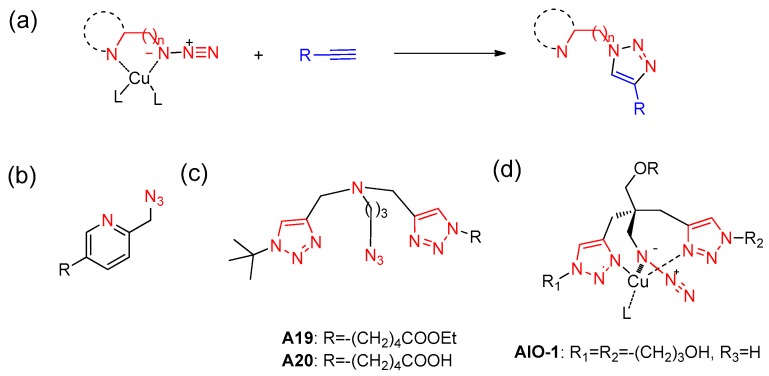
Chelating azides for CuAAC reactions: (**a**) chelation-assisted CuAAC; (**b**) 2-picolylazide; (**c**) A19 and A20; (**d**) AIO-1.

The second strategy for optimizing CuAAC is to develop chelating azides as more reactive substrates (Scheme 4a). Since it is speculated the Cu(I)-azide association is the rate-determining step in the CuAAC catalytic cycle [61,62], the development of chelating azides aims at enhancing this weakest link to acquire the reaction rate acceleration.

The phenomenon that chelating azides can accelerate CuAAC was first evaluated by the Zhu group in 2009 after they discovered that azides containing a nitrogen-based auxiliary ligand such as a pyridine ring next to the azido group could accelerate CuAAC (Scheme 4b) [111]. Nitrogen donor ligands of sp^2^ or sp^3^ hybridization showed no significant difference in CuAAC acceleration, but oxygen or sulfur donors have no such effect [112]. Crystal structures of complexes between 2-picolylazide or 2-(2-azidoethyl)pyridine with CuCl_2_ both revealed the chelation of Cu(II) by the pyridine *N* atom and the alkylated *N* of the azido group [111,112]. Besides, additional addition of TBTA ligand to the reaction system could further facilitate the CuAAC for these chelating azides [112].

In a further mechanism study, they proposed that the high reactivity of chelating azides came from the rapid copper-azido group interaction which occurred prior to Cu(I) acetylide formation, and this rendered the deprotonation of alkyne the rate-determining step [113]. Cu(OAc)_2_ was found to be the most suitable copper source, and this was attributed to its optimal Cu-Cu distance in the dinuclear copper(II) core that could mediate both the alkyne oxidative homocoupling and the CuAAC reactions [113]. Based on these observations, the Zhu group proposed a mechanistic model with a copper(II)/copper(I) mixed valency dinuclear species in the chelation assisted CuAAC of 2-picolylazide [113].

The other branch of chelation-assisted CuAAC uses triazole rings as the chelating moieties. Early in 2005, it was reported that CuAAC of diazide 2,2-bis(azidomethyl)propane-1,3-diol afforded the ditriazole product as the major product, even when the amount of diazide was exceedingly excessive. However, the reaction of dialkyne yielded statistically expected quantities of bi- and mono-triazole products [36]. This phenomenon suggested that an adjacent triazole could accelerate CuAAC reactions by copper chelation.

Based on the tetradentate structure of TBTA, the Taran group developed new chelating azides with a complete copper-chelating system in their structures (Scheme 4c) [114]. These azides were designed to form strong, active copper complexes and should therefore be considered both as reactant and catalyst in the CuAAC reaction. Kinetic studies revealed their outstanding performance in accelerating CuAAC with observed rate constants as high as 1300 M^−1^·s^−1^ (A20) under their reaction conditions [114].

For A19 and its derivatives, the linkage of two triazole rings and an alkylated azido group saturated the tridentate nitrogen atom. To be used in bioorthogonal reactions, tags for detection or visualization had to be placed on the triazole rings [114]. To preserve the positions on triazole rings for compound property adjustment and to provide a more flexible scaffold for tag conjugation, the Zhang group developed the so called all-in-one (AIO) copper-chelating azide reagents with a tetrahedral carbon atom at the core of the structural skeleton (Scheme 4d) [115]. Kinetic studies showed slightly higher k_obs_ rate constants of AIO azide than A19 under model reaction conditions, and CuAAC reactions assisted by both chelating azides were more than 10^2^ times faster than that assisted by TBTA, THPTA or BTTES [115].

Interesting properties of triazole-based chelating azides (A19, A20, and AIO-1) other than the astonishing accelerated reaction rate have been reported. Both A19 and AIO-1 have advanced cellular protective effects towards cytotoxicity induced by copper ion [114,115]. In the kinetic study, addition of BTTES as a ligand was found to slow obviously down the reaction of A20, which is quite different from the phenomenon whereby TBTA facilitated the CuAAC of 2-picolylazide [112,114]. Halide effect on the chelation-assisted CuAAC of AIO-1 was investigated and no obvious inhibitions were observed with excessive amounts of chloride and bromide ions, which is another guarantee for application in biological systems [115]. Kinetic studies also suggested there should be a different mechanism for this kind of chelation-assisted CuAAC in comparison with ligand-assisted CuAAC. Both A20 and AIO-1 had a first order dependence on copper, with a “threshold” point at a chelating azide/Cu ratio of 1/1 [114,115]. This is quite different from the second order dependence on copper and a ‘threshold’ point at ligand/Cu ratio of 2/1 displayed by most ligands [61]. However, after the ‘threshold’ point, A20 and AIO-1 differ in the Cu dependence. Excessive amount of copper dramatically decreased the reaction rate of A20, while AIO-1 showed an almost zero order dependence on copper when supplied with more than one equivalent of copper [114,115].

### 6.2. Bioorthogonal Applications of Chelation Assisted CuAAC

The ultra-fast kinetics and obvious cellular protective effects have promoted the fast spreading applications of chelation-assisted CuAAC to the labeling and detection of proteins [114,115,116], glycans [115,117] and RNAs [116] on the cell and organism level, demonstrating its vast potential for applications in more challenging and complicated biological systems.

The Ting group was the first to apply chelating azides for bioorthogonal labeling in biological systems. Through enzyme engineering, they conjugated a picolyl azide-containing substrate to LAP fusion proteins on outside cell membrane, and labeled this cell surface protein with fluorophore by chelation-assisted CuAAC with reduced amount of copper and the assistance of THPTA or BTTAA [116]. Utility of picolyl azide-containing substrates in bioorthogonal reactions was also demonstrated by sensitive labeling and detection of metabolically labeled proteins and RNAs in cells [116]. The Wu group applied another picolyl azide derivative for the detection of newly synthesized cell surface glycans in mammalian cells and early zebrafish embryogenesis under the assistance of BTTPS as the ligand [117].

Picolyl azide derivatives react faster and more robust in CuAAC compared with non-chelating azides, but they still need the assistance of ligands when applied as bioorthogonal reagents. This limitation is avoided in the cases of the more potent chelating azides A20 and AIO series. The derivatives of these two chelating azides are qualified bioorthogonal reagents under ligand-free conditions. Since chelating azides are usually added in a complex with copper ions, the dispensation of ligands simplifies the CuAAC to a two-component system. The A20 derivative with a fluorophore was used for tracking tubulin that was tagged by alkyne-paclitaxel inside living cells [114]. AIO reagents with a biotin or a fluorophore or both tags were also competent bioorthogonal reagents under the cellular circumstance. By metabolic insertion of alkyne handles, glycan visualization was achieved both in cells and in living *C. elegans* [115]. Specific protein labeling of HER2 protein by an alkynyl covalent inhibitor derivative was also successfully performed on cell level [115].

## 7. Strain-Promoted Azide-Alkyne Cycloaddition (SPAAC)

**Scheme 5:**
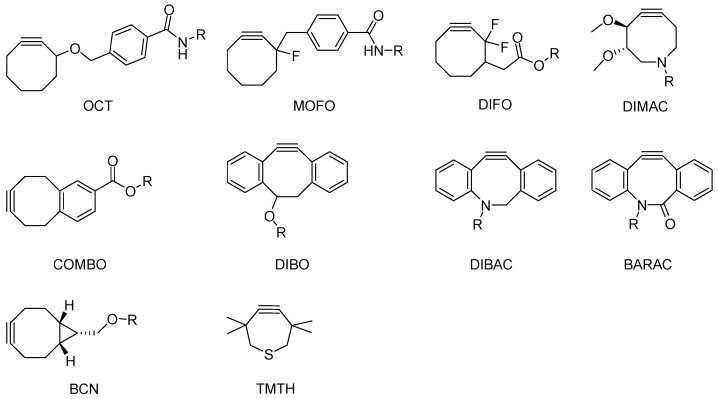
Cyclooctyne derivatives for the SPAAC reaction.

Strain-promoted azide-alkyne cycloaddition (SPAAC) is another strategy developed for optimization of CuAAC. Instead of metal catalysis, the driving force of SPAAC is the ring strain introduced into the alkyne reagents. In 2004, the Bertozzi group developed cyclooctyne (OCT) as the first strain-promoted reactive alkyne for azide-alkyne cycloaddition [118] (Scheme 5). Though the circumvention of cytotoxic Cu ion enhanced the biocompatibility of SPAAC reaction, OCT only had the second-order rate constant k_2_ of 0.0012 M^−1^·s^−1^, which was at the same level as Staudinger reaction, and far slower than CuAAC [9,118].

For the further optimization of SPAAC, various OCT-based derivatives have been developed to improve the second-order rate constant k_2_ [6,9] (Scheme 5). The monofluorinated MOFO and difluorinated DIFO were subsequently developed by the Bertozzi group, and they increased the SPAAC reaction rate by about 40-fold [119,120]. To increase the water solubility, a more hydrophilic cyclooctyne derivative DIMAC was developed by introduction of a nitrogen atom into the ring [121]. DIMAC displayed similar reaction rates as OCT and enhanced biocompatibility. Fusion of one or two benzyl rings to the OCT skeleton was also explored. Monobenzyl-activated COMBO reagents and dibenzyl-activated DIBO and DIBAC reagents further increased the reaction rate [122,123,124,125].

On the basis of DIBAC, movement of the amide from the exocyclic to endocyclic orientation led to the development of BARAC, which has the second-order rate constant k_2_ of 0.96 M^−1^·s^−1^ [126]. Fusion of a cyclopropyl ring (BCN) also improved the reaction rate, though not as much as BARAC [127]. Apart from the OCT skeleton, cyclononyne and cycloheptyne derivatives presented as new approaches. Thiacycloheptyne (TMTH) had the second-order rate constant k_2_ of 4 M^−1^·s^−1^, the fastest SPAAC so far reported [128,129]. However, TMTH couldn’t be equipped with a label, which hinders the further affinity purification or fluorescence detection after bioorthogonal reaction.

SPAAC has been widely applied for bioorthogonal labeling and detection since it avoids the usage of Cu(I) ions. The application examples extend from cell surface labeling to in vivo labeling of various model animals, including zebrafish, *C. elegans* and mice [5,130,131]. However, SPAAC has its own drawbacks [11,130,131]. The major concern is the much slower reaction rate compared with CuAAC. Efforts to improve the kinetics often results in unstable reagents, such as BCN. Another problem is the side reaction of the strained alkynes with thiols, which makes SPAAC not a strictly bioorthogonal reaction. Besides, the poor water solubility and the lengthy yet possibly low yielding synthetic routes are issues to be solved.

## 8. Conclusions

In summary, the past decade has witnessed the booming development of CuAAC, and a marked expansion of its bioorthogonal applications. The discovery of copper catalysis and TBTA as a catalyst ligand have boosted the applications of CuAAC in biological fields. To overcome the major limitation of copper ions induced cytotoxicity and enhance its biocompatibility, different strategies have been investigated, including tailored ligands, chelating azides, and SPAAC. The ligand-assisted CuAAC and chelation-assisted CuAAC have separately optimized the reaction efficiency, reaction speed and biocompatibility issues, making CuAAC more suitable for cellular exploitations. Until today, CuAAC still represents the most competent bioorthogonal toolkit, and it has demonstrated its power on almost all classes of biomolecules, such as proteins, glycans, lipids and nucleic acids. The optimization of CuAAC shall continue in order to assist the exploration of more complicated systems and the monitoring of more sensitive and more dynamic processes.
